# Morphological, molecular and 3D synchrotron X-ray tomographic characterizations of *Helicascus satunensis* sp. nov., a novel mangrove fungus

**DOI:** 10.7717/peerj.18341

**Published:** 2024-11-08

**Authors:** Sita Preedanon, Anupong Klaysuban, Satinee Suetrong, Oraphin Pracharoen, Waratthaya Promchoo, Tanuwong Sangtiean, Catleya Rojviriya, Jariya Sakayaroj

**Affiliations:** 1 National Biobank of Thailand, National Center for Genetic Engineering and Biotechnology (BIOTEC), National Science and Technology Development Agency (NSTDA), Khlong Nueng, Khlong Luang, Pathum Thani, Thailand; 2 Department of Marine and Coastal Resources, Ministry of Natural Resources and Environment, Laksi, Bangkok, Thailand; 3 Synchrotron Light Research Institute (Public Organization), Nakhon Ratchasima, Thailand; 4 School of Science, Walailak University, Nakhon Si Thammarat, Thailand

**Keywords:** Ascomycota, Marine fungi, 3D synchrotron, Helicascus

## Abstract

A new species of *Helicascus satunensis* sp. nov. was collected on mature dead fruits of the *Nypa* palm in Satun Province, southern Thailand. Its morphological characteristics are similar to those of the genus *Helicascus*. Recently, a genus *Helicascus* with three species from marine habitats worldwide was studied. The morphology of this fungus was investigated and combined with multigene sequence analyzes of small subunit (SSU), large subunit (LSU), internal transcribed spacer (ITS) ribosomal DNA, translation elongation factor 1-alpha (TEF-1*α*) and RNA polymerase II (RPB2) genes. Morphologically, *H. satunensis* sp. nov. is characterized by semi-immersed, lenticular ascomata, multilocules, a bitunicate ascus and smooth, obovoid, dark brown ascospores that are one-septate and unequally two-celled. In addition, 3D visualization using synchrotron X-ray tomography was performed to investigate the interaction between fruiting body and substrata. Molecular phylogeny with multigene revealed that *H. satunensis* sp. nov. belongs to the family Morosphaeriaceae, order Pleosporales, class Dothideomycetes. Furthermore, *H. satunensis* sp. nov. forms a well-supported clade with *Helicascus* species described from marine habitats. Based on the unique morphological and molecular evidence, we propose this fungus, *H. satunensis* sp. nov., as a new species for *Helicascus*.

## Introduction

Kohlmeyer (1969) described the distinct marine ascomycete *Helicascus* obtained from dead prop roots of the mangrove *Rhizophora mangle*. A type species, *Helicascus kanaloanus*, is characterized by an immersed ascostroma composed of multilocules that share a common periphysate ostiole lying under pseudostromatic tissues. The asci are subcylindrical bitunicate and pediculated, and the endoascus is usually coiled at the base. The ascospores are brown to dark brown at maturity and are frequently asymmetrically two-celled with a mucilaginous sheath in this species (Kohlmeyer, 1969).

Since 1991, a number of *Helicascus*-like species have been described from freshwater and marine habitats based on their common morphological characteristics and DNA sequences (Hyde, 1991; Zhang et al., 2013; Zhang et al., 2014; Zhang et al., 2015; Luo et al., 2016; Preedanon et al., 2017; Zhang et al., 2024). However, two new genera, *Aquihelicascus* and *Neohelicascus,* were excluded from the genus *Helicascus* due to their morphological and multigene phylogeny (Dong et al., 2020). *Aquihelicascus* was established to accommodate one new combination and two new species. *Neohelicascus* was introduced to accommodate one new species and seven new combinations (Dong et al., 2020).

Recently, three marine species in the genus *Helicascus* were identified, *H. kanaloanus*, *H. nypae* and *H. mangrovei,* based on morphological and molecular data (Kohlmeyer, 1969; Hyde, 1991; Preedanon et al., 2017). *Helicascus nypae* was found on *Nypa* palm fronds from Southeast Asia. It is characterized as having ascomata with immersed multilocules with a single common central ostiole, bitunicate asci with a long, narrow and coiled endoascus, and unequally two-celled, verrucose ascospores surrounded by a gelatinous sheath (Hyde, 1991). Subsequently, Preedanon et al. (2017) reported *H. mangrovei* obtained from decaying mangrove wood in Thailand. The unique morphological characteristics of *H. mangrovei* include multilocular ascomata semi-immersed under a thick clypeus that forms pseudostromata, clavate pedicellate asci in a hamathecium of cellular pseudoparaphyses, dark brown color at maturity, and unequally two-celled ascospores with one apiculate end.

The aim of this study is to report on novel ascomycete found in Thai mangrove habitats. The microscopic morphology of the fungal fruiting bodies and host tissues was visualized in three dimensions using synchrotron radiation X-ray tomography, which enables high-resolution and non-invasive visualization of internal features without the need for serial sections and staining reagents. This capability is simply unattainable with conventional characterization tools (Friis et al., 2014; Sena et al., 2022; Becher, Sheppard & Grunwaldt, 2023). Furthermore, we provide the molecular phylogeny of the combined SSU, LSU, ITS rDNA, TEF-1α and RPB2 sequences to confirm their taxonomic position.

## Materials and Methods

### Sample collection, isolation, morphological examination, and materials availability

Mature dead *Nypa* palm fruits were collected from mangroves at Mangrove Forest Resource Development Station 36 in Satun Province, southern Thailand (6°54′14.9616″N and 99°41′17.4912″E). Collecting procedure was made as previously described in Preedanon et al. (2017). The collected *Nypa* palm fruits were placed in a sealed plastic Ziploc bag and brought back to the laboratory for further examination. The specimens were washed with natural seawater in order to remove sediment and other debris then kept in a moist plastic box and incubated at room temperature (approximately 25–28 °C). The samples were examined directly under a stereo-zoom microscope for the presence of *H. satunensis* sp. nov. Photographic documentation of the sporulating structures was carried out using the Olympus BX51 and Olympus DP21 software (Olympus, Tokyo). The ascomata specimens were fixed with embedding matrix on stage and cutting sections through a cryostat microtome (MEV; SLEE Mainz, Mainz, Germany). The fresh ascomata of *H. satunensis* sp. nov. were selected for single spore isolation (Vrijmoed, 2000). Spore suspension was diluted and plated on 1.5% seawater corn meal agar (SCMA) medium with the addition of antibiotics (streptomycin sulfate 0.5 g/L, penicillin G 0.5 g/L). The germinated spores were placed onto freshly SCMA medium and incubated at room temperature (approximately 25–28 °C) (Preedanon et al., 2017). Axenic cultures (BCC 83546, BCC 86189, BCC 86190) were then transferred to 1.5% seawater potato dextrose agar (SPDA). Colony characteristics, growth and sporulation were observed and recorded. The type cultures were deposited at the BIOTEC Culture Collection (BCC), Pathum Thani, Thailand. In addition, dried voucher type specimens (BBH 50658, BBH 50659 and BBH 50660) were deposited at BIOTEC Bangkok Herbarium (BBH), Pathum Thani, Thailand.

### Three-dimensional synchrotron X-ray tomography

The microstructure of the fungal fruiting bodies and the outer exocarp of *Nypa* palm fruit was visualized in three dimensions using synchrotron radiation X-ray tomographic microscopy (SRXTM). Prior to the SRXTM experiment, the fresh fungal fruiting bodies samples were fixed with 3% formaldehyde. For tomographic imaging, each sample was placed in a sample holder attached to a brass stub with glue to stabilize the sample during tomography scanning. The SRXTM examination of the samples was carried out at the X-ray tomographic microscopy beamline (BL1.2W: XTM) at the Siam Photon Source Facility, Synchrotron Light Research Institute. The X-ray beam was generated from a 2.2-Tesla multipole wiggler radiation source optimized with a toroidal focusing mirror and filtered with aluminum foils to achieve an average energy of 10 keV. All X-ray projections were acquired with a pixel size of 3.61 µm using an imaging system consisting of a 2X objective lens-coupled microscope (Optique Peter, Lentilly, France), a YAG-Ce scintillator (CRYTUR, Turnov, Czech Republic) and the PCO.edge 5.5 sCMOS camera (Excelitas PCO GmbH, Kelheim, Germany). To enhance fine details of the entire sample, a tomographic volume was reconstructed from enlarged composite projections obtained from multiple scans. Each scan covered 180°with a step of 0.2°, resulting in a dataset. Subsequently, the X-ray projection datasets underwent pre-processing, which included flat-field correction, beam intensity normalization and image stitching. Tomographic reconstruction was performed using Octopus Reconstruction software (TESCAN, Ghent, Belgium). The resulting computed tomographic slices were analyzed using ImageJ (http://rsbweb.nih.gov/ij/) and Fiji (http://fiji.sc/Fiji), and the 3D visualization of the tomographic volume was displayed using Drishti software (Limaye, 2012).

### DNA extraction, PCR amplification and DNA sequencing

Genomic DNA from fungal mycelia was extracted according to the methods of O’Donnell et al. (1997) and Sakayaroj, Pang & Jones (2011). Ribosomal DNA genes (ITS, SSU, LSU) and protein-coding gene sequences (TEF-1α, RPB2) were amplified by polymerase chain reaction (PCR). The ITS rDNA region was amplified with the primer pair ITS4/ITS5 (White et al., 1990), the SSU region with NS1/NS4 (White et al., 1990), the LSU region with LROR/LR5 (Vilgalys & Hester, 1990), the TEF1-α region with EF1-983F/EF1-2218R (Rehner & Buckley, 2005), the RPB2 region with fRPB2-5F/fRPB2-7cR (Liu, Whelen & Hall, 1999) (Table 1). The component of PCR reaction was performed in a total volume of 50 µL, containing 1 µL DNA template (30–50 ng/ µL), 1 µL of each forward and reverse primers (10 µM), 10 µL master mix of Taq DNA polymerase (Thermo Fisher Scientific Inc., Waltham, MA, USA) and 37 µL of double-distilled water. The PCR conditions for all the genes used were set up using the T100TM thermal cycler (BIO-RAD Laboratories, Inc., California) (Table 1). The PCR products were subsequently purified and sequenced by Macrogen (Seoul, South Korea).

**Table 1:** PCR primers and amplification profiles used in this study.

**DNA region**	**Primer name**	**Amplification profile**			**Reference**
		**Denaturation**	**Repeat steps**	**Extension**	
Internal transcribed spacer rDNA (ITS)	ITS5 ITS4	94 °C (2 min)	35 cycles 94 °C (1 min) 54 °C (1 min) 72 °C (2 min)	72 °C (10 min)	White et al. (1990)
18S small subunit rDNA (SSU)	NS1 NS4	94 °C (2 min)	35 cycles 94 °C (1 min) 55 °C (1 min) 72 °C (2 min)	72 °C (10 min)	White et al. (1990)
28S large subunit rDNA (LSU)	LROR LR5	94 °C (2 min)	35 cycles 94 °C (1 min) 55 °C (1.5 min) 72 °C (2.5 min)	72 °C (10 min)	Vilgalys & Hester (1990)
Translation elongation factor 1-alpha (TEF 1-*α*)	EF1-983F EF1-2218R	95 °C (2 min)	35 cycles 95 °C (1 min) 54 °C (1 min) 72 °C (2 min)	72 °C (10 min)	Rehner & Buckley (2005)
RNA polymerase II second largest subunit (RPB2)	fRPB2-5F fRPB2-7cR	94 °C (3 min)	35 cycles 94 °C (1 min) 54 °C (1 min) 72 °C (1.5 min)	72 °C (8 min)	Liu, Whelen & Hall (1999)

**Table 2:** Taxa and sequences database accession numbers used in this study. Newly generated sequences are indicated in bold.

**Taxon**	**Strain**	**GenBank accession no.**				
		**LSU** **rDNA**	**SSU** **rDNA**	**ITS** **rDNA**	**TEF-1α**	**RPB2**
*Aquihelicascus songkhlaensis*	MFLUCC 18-1154^T^	MN913692	–	MT627680	MT954380	–
*Aquihelicascus songkhlaensis*	MFLUCC 18-1273	MN913724	MT864319	MT627696	MT954369	MT878464
*Aquihelicascus songkhlaensis*	MFLUCC 18-1278	MN913726	MT864318	MT627693	MT954366	MT878458
*Aquihelicascus thalassioideus*	MFLUCC 10-0911^T^	KC886636	KC886637	KC886635	–	–
*Aquihelicascus thalassioideus*	MJF 14020-2	KP637165	–	KP637162	–	–
*Aquihelicascus thalassioideus*	JCM 17526	AB807558	AB797268	LC014554	AB808534	–
*Aquihelicascus thalassioideus*	CBS 110441	AB807557	AB797267	LC014553	AB808533	–
*Aquihelicascus thalassioideus*	KUMCC 19-0094	MT627668	–	MT627689	–	–
*Aquihelicascus yunnanensis*	MFLUCC 18-1025^T^	MN913711	MT864292	MT627728	MT954391	–
*Aquilomyces patris*	CBS 135661^T^	KP184041	KP184077	KP184002	–	–
*Aquilomyces patris*	CBS 135760	KP184042	KP184078	KP184004	–	–
*Aquilomyces patris*	CBS 135662	KP184043	KP184079	KP184003	–	–
*Aquilomyces rebunensis*	CBS 139684^T^	AB807542	AB797252	AB809630	AB808518	–
*Clypeoloculus akitaensis*	CBS 139681^T^	AB807543	AB797253	AB809631	AB808519	–
*Clypeoloculus hirosakiensis*	CBS 139682^T^	AB807550	AB797260	AB809638	AB808526	–
*Clypeoloculus microsporus*	CBS 139683^T^	AB807535	AB797245	AB811451	AB808510	–
*Clypeoloculus towadaensis*	CBS 139685^T^	AB807549	AB797259	AB809637	AB808525	–
*Didymella fucicola*	JK 2932	EF177852	–	EF192138	–	–
*Falciformispora lignatilis*	BCC 21118	GU371827	GU371835	–	GU371820	–
*Halojulella avicenniae*	BCC 18422	GU371823	GU371831	–	GU371816	GU371787
*Halojulella avicenniae*	BCC 20173	GU371822	GU371830	–	GU371815	GU371786
*Halojulella avicenniae*	JK 5326A	GU479790	GU479756	–	–	–
*Helicascus kanaloanus*	A 237	–	AF053729	–	–	–
*Helicascus kanaloanus*	ATCC 18591	KX639748	KX639744	KX957961	KX639756	KX639752
*Helicascus mangrovei*	BCC 68258^T^	KX639745	KX639741	KX957958	KX639753	KX639749
*Helicascus mangrovei*	BCC 68260	KX639746	KX639742	KX957959	KX639754	KX639750
*Helicascus mangrovei*	BCC 74471	KX639747	KX639743	KX957960	KX639755	KX639751
*Helicascus nypae*	BCC 36751	GU479788	GU479754	–	GU479854	GU479826
*Helicascus nypae*	BCC 36752	GU479789	GU479755	–	GU479855	GU479827
***Helicascus satunensis***	**BCC 83546** ^**T**^	**PP866393**	**PP873998**	**PP873995**	**PP915719**	**PP915722**
***Helicascus satunensis***	**BCC 86189**	**PP866394**	**PP873999**	**PP873996**	**PP915720**	**-**
***Helicascus satunensis***	**BCC 86190**	**PP866395**	**PP874000**	**PP873997**	**PP915721**	**PP915723**
*Leptosphaeria maculans*	AFTOL ID-277	DQ470946	DQ470993	–	DQ471062	DQ470894
*Massarina igniaria*	CBS 845.96	DQ810223	DQ813511	–	–	–
*Microvesuvius unicellularis*	AD 291626	OQ799383	–	OQ799384	OQ866586	–
*Microvesuvius unicellularis*	AD 291633^T^	OQ799391	–	OQ799382	OQ866585	–
*Montagnula opulenta*	AFTOL ID-1734	DQ678086	AF164370	–	–	DQ677984
*Morosphaeria muthupetensis*	PUFD 87^T^	MF614796	MF614797	MF614795	MF614798	–
*Morosphaeria ramunculicola*	BCC 18404	GQ925853	GQ925838	–	–	–
*Morosphaeria ramunculicola*	BCC 18405	GQ925854	GQ925839	–	–	–
*Morosphaeria ramunculicola*	JK 5304B	GU479794	GU479760	–	–	GU479831
*Morosphaeria ramunculicola*	KH 220	AB807554	AB797264	–	AB808530	–
*Morosphaeria velatispora*	BCC 17059	GQ925852	GQ925841	–	–	–
*Morosphaeria velatispora*	NBRC 107812	AB807556	AB797266	LC014572	AB808532	–
*Neohelicascus aegyptiacus*	MFLU 12-0060^T^	KC894853	KC894852	–	–	–
*Neohelicascus aquaticus*	KUMCC 19-0107	MT627662	MT864314	MT627719	MT954384	–
*Neohelicascus aquaticus*	KUMCC 17-0145	MG356477	MG356487	MG356479	MG372317	–
*Neohelicascus aquaticus*	MFLUCC 17-2300	MG356478	–	MG356480	MG372316	–
*Neohelicascus aquaticus*	MFLUCC 10-0918^T^	KC886640	KC886638	KC886639	MT954384	–
*Neohelicascus aquaticus*	MAFF 243866	AB807532	AB797242	AB809627	AB808507	–
*Neohelicascus chiangraiensis*	MFLUCC 13-0883^T^	KU900585	KU900587	KU900583	KX455849	–
*Neohelicascus elaterascus*	MAFF 243867	AB807533	AB797243	AB809626	AB808508	–
*Neohelicascus elaterascus*	CBS 139689	LC014608	LC014603	LC014552	LC014613	–
*Neohelicascus elaterascus*	MFLUCC 18-0985	MT627658	MT864335	MT627735	–	–
*Neohelicascus elaterascus*	MFLUCC 18-0993	MT627659	MT864333	MT627730	–	–
*Neohelicascus elaterascus*	HKUCC 7769	AY787934	AF053727	–	–	–
*Neohelicascus gallicus*	BJFUCC 200228	KM924831	–	KM924833	–	–
*Neohelicascus gallicus*	CBS 123118	KM924832	–	–	–	–
*Neohelicascus gallicus*	BJFUCC 200224	KM924830	–	–	–	–
*Neohelicascus griseofavus*	MFLUCC 16-0869^T^	OP377964	OP378041	OP377878	OP473055	–
*Neohelicascus submersus*	MFLU 20-0436^T^	MT627656	MT864340	MT627742	–	–
*Neohelicascus unilocularis*	MJF 14020^T^	KP637166	–	KP637163	–	–
*Neohelicascus unilocularis*	MJF 14020-1	KP637167	–	KP637164	–	–
*Neohelicascus uniseptatus*	MFLUCC 15-0057^T^	KU900584	–	KU900582	KX455850	–
*Paradendryphiella arenariae*	AFTOL ID-995^T^	DQ470971	DQ471022	–	DQ677890	DQ470924
*Parastagonospora avenae*	AFTOL ID-280	AY544684	AY544725	–	DQ677885	DQ677941
*Phaeodothis winteri*	AFTOL ID-1590	DQ678073	DQ678021	–	DQ677917	DQ677970
*Phaeosphaeria eustoma*	AFTOL ID-1570	DQ678063	DQ678011	–	DQ677906	DQ677959
*Platychora ulmi*	CBS 361.52	EF114702	EF114726	–	–	–
*Plenodomus biglobosus*	CBS 303.51	GU301826	–	–	GU349010	–
*Setoseptoria arundinacea*	CBS 619.86	DQ813509	DQ813513	–	–	–
*Stemphylium vesicarium*	CBS 191.86^T^	DQ247804	DQ247812	–	DQ471090	DQ247794
*Trematosphaeria pertusa*	CBS 122371	FJ201992	FJ201993	–	–	–
**Outgroup**						
*Lophiostoma macrostomum*	JCM 13544	AB619010	AB618691	JN942961	LC001751	JN993491
*Sigarispora arundinis*	JCM 13550	AB618998	AB618679	JN942964	LC001737	JN993482

**Notes.**

^T^ Ex-type strain

### Phylogenetic analyses

Multiple sequence alignments were analyzed with the closely matched sequences obtained from GenBank (Table 2) according to Jones et al. (2015), Maharachchikumbura et al. (2016) and Hongsanan et al. (2017), Dong et al. (2020), Yang et al. (2023a) and Yang et al. (2023b). The newly generated sequences from this study are listed in Table 2. The nucleotide sequences were assembled and aligned using BioEdit 7.2.5 (Hall, 1999) and Muscle 3.8.31 (Edgar, 2004). Specifically, NCBI blast searches were used to determine sequence similarity to sequences published in the GenBank database. Phylogenetic analyses of the combined SSU, LSU, ITS rDNA, TEF-1α and RPB2 sequences were performed using maximum likelihood (ML) and Bayesian algorithms. Maximum likelihood (ML) analysis was evaluated in RAxMLHPC2 on XSEDE (Stamatakis, 2014) *via* the CIPRES Science Gateway platform (Miller, Pfeiffer & Schwartz, 2010) under the GTR + GAMMA model with BFGS method to optimize the GTR rate parameters. Finally, Bayesian posterior probabilities of branches were performed using MrBayes 3.2.6 (Ronquist et al., 2012), with the best-fitting model (GTR+I+G) selected by AIC in MrModeltest 2.2 (Nylander, 2004), which was tested with hierarchical likelihood ratios (hLRTs). Three million generations were run in four Markov chains and a sample was drawn every 100 generations with a burn-in value of 3,000 sampled trees. Finally, the consensus tree was displayed using the interactive Tree Of Life (iTOL) (Letunic & Bork, 2021) and adjusted in Adobe Photoshop 2020. All sequences obtained in this study were submitted to GenBank, and the typification were published in the MycoBank database (Crous et al., 2004). The resulting alignments were submitted to TreeBASE (submission numbers: 31389).

### Nomenclature

The electronic version of this article in Portable Document Format (PDF) will represent a published work according to the International Code of Nomenclature for algae, fungi, and plants, and hence the new names contained in the electronic version are effectively published under that Code from the electronic edition alone, so there is no longer any need to provide printed copies. In addition, new names contained in this work have been submitted to MycoBank from where they will be made available to the Global Names Index. The unique MycoBank number can be resolved and the associated information viewed through any standard web browser by appending the MycoBank number contained in this publication (MB 854336) to the prefix http://www.mycobank.org/MB/. The online version of this work is archived and available from the following digital repositories: PeerJ, PubMed Central SCIE, and CLOCKSS.

## Results

### Taxonomy

**Table:** 

**Figure 1:**
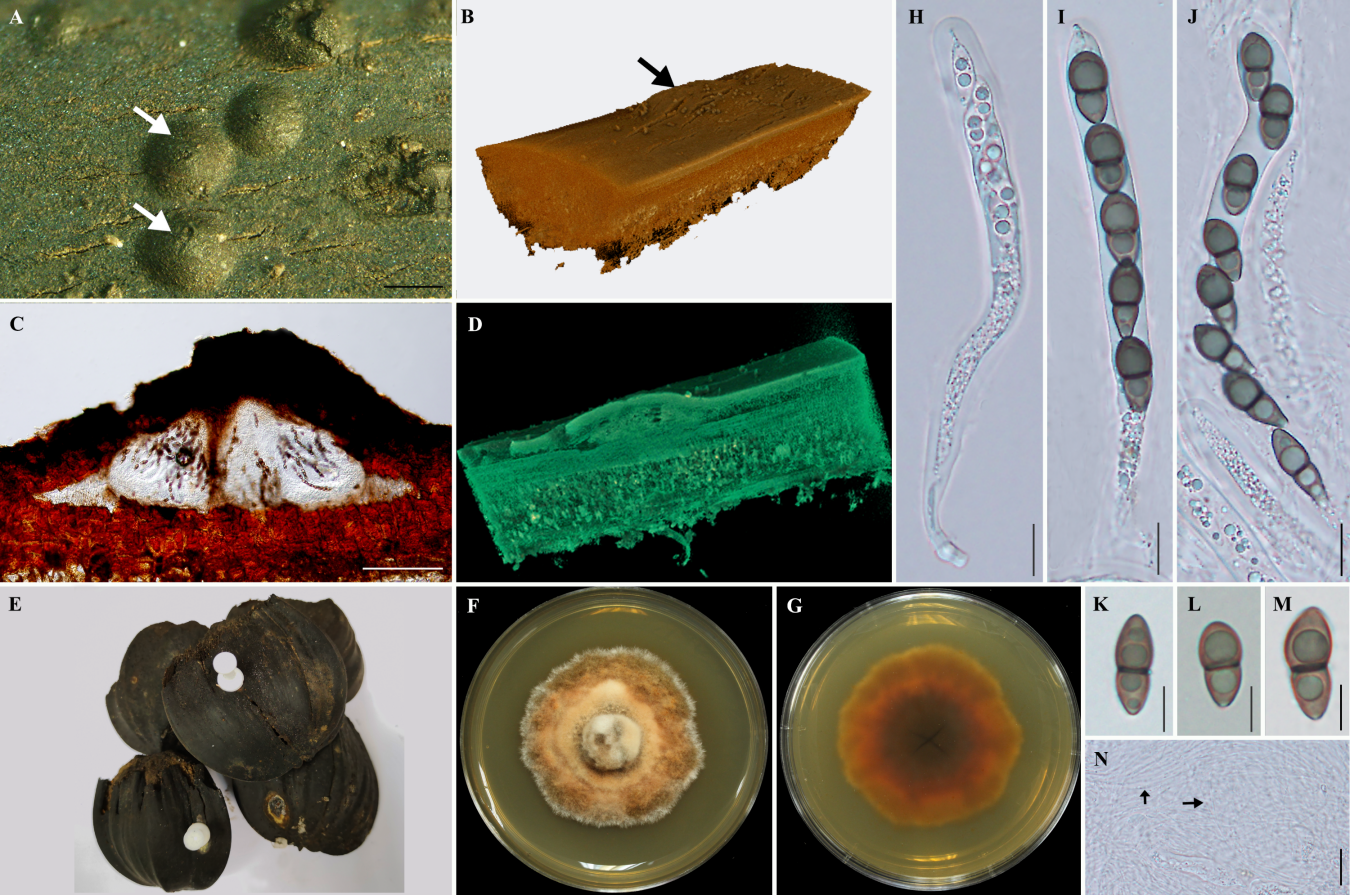
Morphological features of *Helicascus satunensis* sp. nov. (A) Carbonaceous ascomata on the exocarp of *Nypa* palm fruit. (B) The 3D visualization of the ascoma by X-ray tomography (arrow). (C) Vertical section through an ascoma. (D) Section through an ascoma using 3D visualization. (E) Dead *Nypa* palm fruits. (F–G) Obverse and reverse views of cultures grown on SPDA after 60 days. (H–J) Subcylindrical bitunicate asci. (K–M) Ascospores. (N) Pseudoparaphyses (arrow). Scale bars *A* = 1 mm, C =300 µm, H–J = 100 µm, K–M = 10 µm, *N* = 20 µm.

***Helicascus satunensis* Preedanon, Suetrong & Sakay., sp. nov.**Fig. 1.
**MycoBank** ** (MB#** **854336)**
**GenBank** (**SSU rDNA**=PP873998, ** LSU rDNA**=PP866393, ** ITS rDNA**=PP873995, ** TEF-1α**=PP915719, ** RPB2**=PP915722)

**Type**: THAILAND, Satun Province, mangrove forests, on a piece of dead palm (*Nypa fruticans*) fruit, 22 December 2016, S. Preedanon, A. Klaysuban, O. Pracharoen & J. Sakayaroj, BBH 50658*, holotypus, cultura dessicata*, (holotype designated here) (BIOTEC Bangkok Herbarium, Pathum Thani, Thailand).

Ex-type culture: MCR 00699 (BCC 83546) (BIOTEC Culture Collection, Pathum Thani, Thailand).

**Etymology**: ‘satunensis’ referring to the collecting location, Satun Province, southern Thailand, where the fungus was collected.

**Sexual morph**: Ascomata 1,000–2, 400 × 160–280 µm, semi-immersed, lenticular ascomata, 3–4 locules, dark brown to black, carbonaceous, solitary (Fig. 1).

Three-dimensional synchrotron X-ray tomographic analysis reveals that the fungal tissues growing in the outer exocarp of *Nypa* palm fruits, enclosing 3–4 locules with flattened base, horizontally arranged under the pseudostroma. Cellular, numerous, persistent, hyaline pseudoparaphyses.

Asci 475–642. 5 × 62.5–80 µm, 8-spored, bitunicate asci, cylindrical, thick-walled, short hook pedunculate, with an ocular chamber. Ascospores 22.5–25 × 5–8.75 µm, unequally two-celled, smooth, dark-brown, and slightly constricted at the septum, thick-walled (Fig. 1).

**Habitat and distribution**: mangrove forests, Satun Province, southern Thailand.

**Asexual morph**: Undetermined

**Culture characteristics**: Ascospores germinated on SCMA after 1–2 days, colonial grown on SPDA attaining 2–3 cm in diameter after 60 days incubation at room temperature (approximately 25–28 °C), dense, circular, irregular and grey (7D1) with orange patches (7C5) in the center, white (7A1) at the edge; dark brown at reverse side. Colour codes in the fungal description follow “Methuen Handbook of Colour” (Kornerup & Wanscher, 1978).

### Phylogenetic analyses

**Figure 2:**
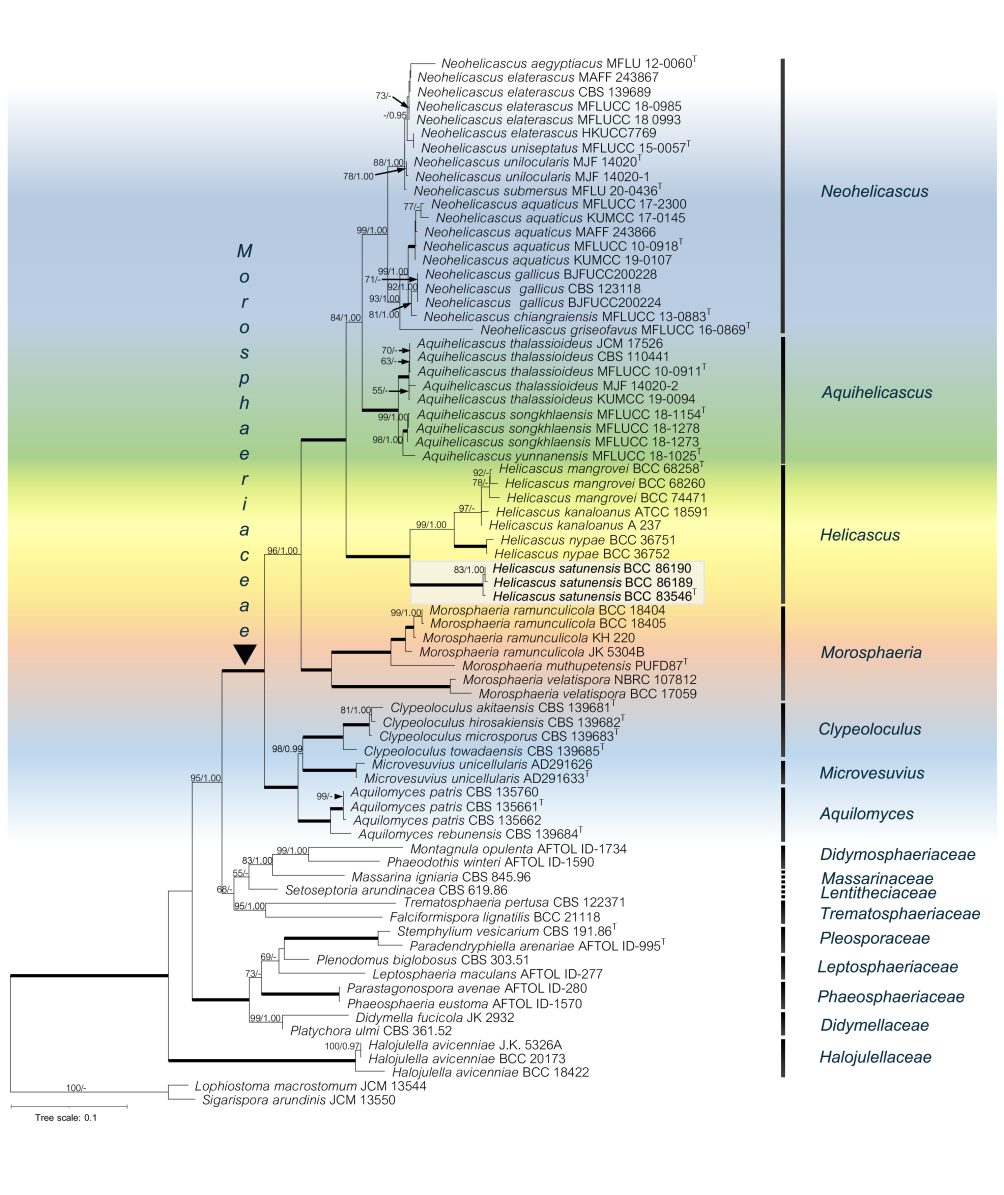
The RAxML phylogenetic tree of *Helicascus satunensis* (BCC 83546, BCC 86189, BCC 86190) resulted from the combined of LSU, SSU, ITS, TEF-1α and RPB2 sequences. *Lophiostoma macrostomum* and *Sigarispora arundinis* were used as outgroups. Maximum likelihood (BSML, left) equal to or greater than 50% are shown above each branch. Bayesian posterior probabilities (BYPP, right) equal to or greater than 0.95 are shown below each branch. The nodes that are strongly supported by bootstrap proportions (100%) and posterior probabilities (1.00) are shown in a thicker line. Abbreviations: T = ex-type. Novel species is demonstrated in bold.

The phylogenetic relationships of the Pleosporales, Dothideomycetes were reconstructed using the combined five-gene dataset (SSU, LSU, ITS rDNA, TEF-1α, RPB2), with *Lophiostoma macrostomum* JCM 13544 and *Sigarispora arundinis* JCM 13550 as the outgroups (Table 2). The alignment of 74 taxa comprised 5,844 base pairs (1,342 for SSU, 1,358 for LSU, 1,192 for ITS, 968 for TEF-1*α* and 984 for RPB2). Total 3,825 characters were constant; 1,570 characters were parsimony-informative and 449 variable characters were parsimony-uninformative. Phylogenetic analyses showed that our novel species (in bold) belongs to the Morosphaeriaceae. Although the topology of the BI tree and the exhibited ML tree are comparable, the BI tree is not shown. The phylogenetic trees representing the unique position of other species in the marine habitat were deposited in MycoBank. Significant ML bootstrap values (≥50%) and Bayesian posterior probabilities (≥0.95) are indicated in the phylogenetic tree (Fig. 2).

In the multigene phylogenetic analysis, the Morosphaeriaceae are divided into subclades representing the seven accepted genera including *Neohelicascus, Aquihelicascus, Helicascus, Morosphaeria, Clypeoloculus, Microvesuvius,* and *Aquilomyces*. Our fungal strains (BCC 83546, BCC 86189 and BCC 86190) are monophyletic and well placed in the Morosphaeriaceae with robust bootstrap and Bayesian supports. They are phylogenetically distinct from the type species *H. kanaloanus* and form sister subclades with *H. nypae* and *H. mangrovei* (Fig. 2). Within the *Helicascus* subclade, we compared the base substitutions of our new fungus with the type species *H. kanaloanus*. The result shows the base substitutions at several sites of SSU (960/969 = 99.0% similarity), LSU (803/853 = 94.1% similarity), ITS rDNA (448/714 = 62.7% similarity), TEF-1 α (872/933 = 93.4% similarity) and RPB2 (793/899 = 88.2% similarity) (Table 3).

## Discussion

### Taxonomy

Jones et al. (2022) reported 1,900 marine fungi in 769 genera that have evolved for marine life as saprobes, parasites and endophytes. Devadatha et al. (2021) and Zhang et al. (2024) reported that many new taxa have been described from mangrove trees and salt marsh plants. Among these host plants, palms found in mangroves and estuaries, such as *Phoenix paludosa, Oncosperma tigillarium*, and *N. fruticans*, harbour a great diversity of fungi (Zhang et al., 2024).

Some of the fungi are found as saprobes on the petioles of *N. fruticans*: *Bacusphaeria nypae*, *Manglicola guatemaelensis,* and *Tirisporella baccariana* (Jones et al., 1996; Suetrong et al., 2009; Abdel-Wahab et al., 2017). *Acuminatispora palmarum*, *Fasciatispora nypae, Helicascus nypae, Neomorosphaeria mangrovei*, *Pleurophomopsis nypae, Striatiguttula nypae,* and *S . phoenicis* grow on submerged rachis and petioles of *N. fruticans* and *Ph. paludosa* (Hyde et al., 1999; Hyde & Alias, 2000; Loilong et al., 2012; Zhang et al., 2018; Zhang et al., 2019; Zhang et al., 2024). A few fungi, however, were discovered on *Nypa* fruits: *Anthostomella nypae*., *Fasciatispora* spp., and *Vaginatispora nypae* (Jayasiri et al., 2019; Zhang et al., 2024).

**Table 3:** Pairwise DNA comparison of *H. satunensis* sp. nov. with the *Helicascus* species.

**DNA sequence**	**Number of bases for comparison** **(bp)**	***Helicascus kanaloanus***		***H. nypae***		***H. mangrovei***	
		**Base substitutions**	**%** **similarity**	**Base substitutions**	**%** **similarity**	**Base substitutions**	**%** **Similarity**
SSU rDNA	969	9	99.0	14	98.5	9	99.0
LSU rDNA	853	50	94.1	47	94.5	40	95.3
ITS rDNA	714	266	62.7	ND	ND	265	62.9
TEF-1α	933	61	93.4	74	92.0	64	93.1
RPB2	899	106	88.2	115	87.2	107	88.1

**Notes.**

ND Not determined

The genus *Helicascus* is a distinct marine ascomycete characterized by having a pseudostroma composed of host cells enclosed in fungal hyphae, subcylindrical asci, uniseriate, obovoid, dark brown color at maturity, and asymmetrical ascospores (Kohlmeyer, 1969). Recently, three species have been identified in the genus, namely, *H. kanaloanus* (type species), *H. nypae*, and *H. mangrovei* (Kohlmeyer, 1969; Hyde, 1991; Preedanon et al., 2017). We found a new fungus, *H. satunensis*, that inhabits the brackish waters of *Nypa* palm fruit in Satun Province, southern Thailand. *Helicascus satunensis* shares similar ascostromata with *H. kanaloanus* and *H. nypae* in having semi-immersed or immersed, carbonaceous, multilocules in the ascostromata arranged under a black pseudoclypeus, while *H. mangrovei* does not have separate locules in the ascomata (Table 4).

**Table 4:** Morphological comparison among species of *Helicascus* (Kohlmeyer, 1969; Hyde, 1991; Preedanon et al., 2017; Zhang et al., 2024).

	***H*** ***elicascus*** ***kanaloanus***	***H. nypae***	***H. mangrovei***	***H. satunensis*** ** sp. nov.**
**Pseudostromata**				
Size (µm)	600–780 × 1,250–2,750	260–390 × 750–1,500	1,500–1, 750 × 1,500–2,500	1,000–2, 400 × 160–280
Position on substrata	Immersed	Immersed	Semi-immersed	Semi-immersed
Locules	Multilocules (3–4 loculi)	Multilocules (3–4 loculi)	Single locule	Multilocules (3–4 loculi)
Structure	Ampulliform, lenticular, horizontally arranged under a black pseudoclypeus	Lenticular, black, carbonaceous	Lenticular, flattened, carbonaceous, solitary, a locule covered by a pseudoclypeus	Lenticular, black, carbonaceous, solitary
**Asci**				
Size (µm)	200–260 × 15–25	192–280 × 14–20	400–412. 5 × 25–30	475–642. 5 × 62.5–80
Shape	Subcylindrical to oblong clavate, persistent, pedunculate, thick-walled	Subcylindrical, pedunculate	Subcylindrical, pedunculate, thick-walled	Cylindrical, thick-walled
Endoascus	With an apical apparatus coiling	With an ocular chamber	With an apical apparatus coiling	Short hook pedunculate with an ocular chamber
**Pseudoparaphyses**	Cellular, numerous, persistent	Cellular, numerous, persistent, anastomosing in a gel	Cellular, numerous, trabeculate, hyaline	Cellular, numerous, persistent, hyaline
**Ascospores**				
Size (µm)	30–55 × 17–25	25–35 × 12 –15	40–45 × 18.5–20	22.5–25 × 5–8.75
Sheath	Present in some collection	Present	Absent	Absent
Shape	Obovoid, brown, biseriate one-septate, constricted at the septum, dark-brown at maturity, unequally two-celled	Uniseriate, obovoid, constricted at the septum, brown, sometimes at one or both ends apiculate, unequally two-celled	Uniseriate, obovoid, unequally two-celled, slightly constricted at the septum, thick-walled and only one apiculate end, dark brown at maturity	Constricted at the septum, thick-walled, unequally two-celled, dark brown at maturity
Ornamentation	Smooth wall	Verrucose wall	Smooth wall	Smooth wall
**Host**	Dead mangrove wood	*Nypa fruticans, Phoenix paludosa* fronds	Dead mangrove wood	*Nypa fruticans* fruit
**Asexual morph**	Undetermined	*Pleurophomopsis nypae*	Undetermined	Undetermined

Pseudostroma is a unique taxonomic characteristic of the Morosphaeriaceae at the genus level. Zhang et al. (2015) reported that multilocular pseudostroma are important in delineating species of *Helicascus*-like species. Members of the Morosphaeriaceae develop somatic hyphae into ascostroma, which subsequently form locules that include the genera *Helicascus, Neohelicascus, Aquihelicascus, Morosphaeria, Clypeoloculus*, and *Aquilomyces* (Dong et al., 2020). The coiling and stretching mechanism of the basal endoascus with an ocular chamber are regarded as unique types of asci in the genus *Helicascus*. Our new fungus *H. satunensis* shares this type of asci with three other species in the genus (Zhang et al., 2015). All species share the same arrangement of cellular pseudoparaphyses. The ascospores of *H. satunensis* can be distinguished from those of other species because they are smaller (22.5–25 × 5–8.75 µm) than those of *H. kanaloanus* (30–55 × 17–25 µm), *H. nypae* (25–35 × 12–15 µm), and *H. mangrovei* (40–45 × 18.5–20 µm). Germ pores were not observed in *H. satunensis*, while they appeared at only one end in *H. mangrovei* (Preedanon et al., 2017). Moreover, the unequal number of *H. satunensis* 2-cell cones with constriction of ascospores could be a defined taxonomic marker at the species level in the genus *Helicascus*.

### Molecular phylogeny

Phylogenetic analyses of multigene sequences revealed that *Helicascus satunensis* forms a well-supported clade within the Morosphaeriaceae, Pleosporales, Dothideomycetes. The Morosphaeriaceae family was established by Suetrong et al. (2009) based on morphological features and strong phylogenetic support. Currently, the family comprises eight genera: *Aquihelicascus* (Dong et al., 2020), *Aquilomyces* (Knapp et al., 2015), *Clypeoloculus* (Tanaka et al., 2015), *Helicascus* (Kohlmeyer, 1969; Dong et al., 2020), *Microvesuvius* (Fryar, Réblová & Catcheside, 2023), *Morosphaeria* (Suetrong et al., 2009), *Neohelicascus* (Dong et al., 2020), and *Neomorosphaeria* (Zhang et al., 2024).

Members in the Morosphaeriaceae family are found on submerged dead twigs in freshwater and marine environments. The multigene phylogeny comprising freshwater taxa formed sister clades to the marine fungal lineages. Two new freshwater genera, *Aquihelicascus* and *Neohelicascus,* were excluded from the genus *Helicascus* due to morphological and molecular evidence (Dong et al., 2020). *Aquihelicascus* was established to accommodate one new combination (*A. thalassioideus*) and two new species (*A. songkhlaensis* and *A. yunnanensis*). *Neohelicascus* was introduced to accommodate one new species (*N. submersus*) and seven new combinations (*N. elaterascus, N. chiangraiensis, N. unilocularis, N. uniseptatus, N. aegyptiacus, N. gallicus,* and *N. aquaticus*) (Dong et al., 2020). The genera *Morosphaeria* (*M. ramunculicola, M. muthupetensis, M. velatispora*) (Suetrong et al., 2009; Devadatha et al., 2018), *Neomorosphaeria* (Zhang et al., 2024), and *Helicascus* (*H. kanaloanus, H. nypae, H. mangrovei*) are predominant saprobic on decaying mangroves and marine substrata, while only *Aquilomyces patris* is a root endophyte of white poplar (Fryar, Réblová & Catcheside, 2023).

The multigene phylogeny in the present study showed that our new fungus *H. satunensis* forms a distinct lineage within the genus *Helicascus* with robust statistical support (100% ML bootstrap and 1.00 Bayesian posterior probability). The DNA sequences of *H. satunensis* differ from those of *H. kanaloanus* and other species in terms of the number of nucleotide base substitutions in all the DNA regions, which indicates that these species are different. In conclusion, with its unique morphological and multigene phylogeny, we introduce *H. satunensis* as a novel mangrove fungus.

##  Supplemental Information

10.7717/peerj.18341/supp-1 Supplemental Information 1 Multigene DNA sequence alignment

10.7717/peerj.18341/supp-2 Supplemental Information 2 SSU sequences for GenBank submission

10.7717/peerj.18341/supp-3 Supplemental Information 3 LSU sequences for GenBank submission

10.7717/peerj.18341/supp-4 Supplemental Information 4 ITS sequences for GenBank submission

10.7717/peerj.18341/supp-5 Supplemental Information 5 TEF sequences for GenBank submission

10.7717/peerj.18341/supp-6 Supplemental Information 6 RPB2 sequences for GenBank submission

## Additional Information and Declarations

### DNA Deposition

The following information was supplied regarding the deposition of DNA sequences: The DNA Sequences are available at GenBank: PP866393, PP873998, PP873995, PP915719, PP915722, PP866394, PP873999, PP873996, PP915720, PP866395, PP874000, PP873997, PP915721, PP915723.

### Data Availability

The following information was supplied regarding data availability: MycoBank number contained in this publication MB# 854336. http://www.mycobank.org/MB/. The Mycobank submission sheet is available in the Supplement File.

### New Species Registration

The following information was supplied regarding the registration of a newly described species: Helicascus satunensis Preedanon, Suetrong & Sakay., sp. nov. MycoBank (MB#854336) http://www.mycobank.org/MB/
The type cultures were deposited at the BIOTEC Culture Collection (BCC), Pathum Thani, Thailand. In addition, dried voucher type specimens (BBH 50658, BBH 50659 and BBH 50660) were deposited at BIOTEC Bangkok Herbarium (BBH), Pathum Thani, Thailand.
